# Developing a model to predict unfavourable treatment outcomes in patients with tuberculosis and human immunodeficiency virus co-infection in Delhi, India

**DOI:** 10.1371/journal.pone.0204982

**Published:** 2018-10-03

**Authors:** Chandravali Madan, Kamal Kishore Chopra, Srinath Satyanarayana, Diya Surie, Vineet Chadha, Kuldeep Singh Sachdeva, Ashwani Khanna, Rajesh Deshmukh, Lopamudra Dutta, Amit Namdeo, Ajay Shukla, Karuna Sagili, Lakhbir Singh Chauhan

**Affiliations:** 1 Evidence Action- Deworm the World Initiative, New Delhi, India; 2 New Delhi Tuberculosis Centre, New Delhi, India; 3 International Union Against Tuberculosis and Lung Disease, New Delhi, India; 4 Division of Global HIV and TB, Centers for Disease Control and Prevention, Atlanta, Georgia, United States of America; 5 National Tuberculosis Institute, Bangalore, Karnataka, India; 6 National AIDS Control Organisation, New Delhi, India; 7 Lok Nayak Chest Clinic, New Delhi, India; 8 The United Nations Children's Fund (UNICEF), New Delhi, India; 9 Uttar Pradesh State AIDS Control Society, Lucknow, Uttar Pradesh, India; 10 Tuberculosis Association of India, New Delhi, India; The Foundation for Medical Research, INDIA

## Abstract

**Background:**

Tuberculosis (TB) patients with human immunodeficiency virus (HIV) co-infection have worse TB treatment outcomes compared to patients with TB alone. The distribution of unfavourable treatment outcomes differs by socio-demographic and clinical characteristics, allowing for early identification of patients at risk.

**Objective:**

To develop a statistical model that can provide individual probabilities of unfavourable outcomes based on demographic and clinical characteristics of TB-HIV co-infected patients.

**Methodology:**

We used data from all TB patients with known HIV-positive test results (aged ≥15 years) registered for first-line anti-TB treatment (ATT) in 2015 under the Revised National TB Control Programme (RNTCP) in Delhi, India. We included variables on demographics and pre-treatment clinical characteristics routinely recorded and reported to RNTCP and the National AIDS Control Organization. Binomial logistic regression was used to develop a statistical model to estimate probabilities of unfavourable TB treatment outcomes (i.e., death, loss to follow-up, treatment failure, transfer out of program, and a switch to drug-resistant regimen).

**Results:**

Of 55,260 TB patients registered for ATT in 2015 in Delhi, 928 (2%) had known HIV-positive test results. Of these, 816 (88%) had drug-sensitive TB and were ≥15 years. Among 816 TB-HIV patients included, 157 (19%) had unfavourable TB treatment outcomes. We developed a model for predicting unfavourable outcomes using age, sex, disease classification (pulmonary versus extra-pulmonary), TB treatment category (new or previously treated case), sputum smear grade, known HIV status at TB diagnosis, antiretroviral treatment at TB diagnosis, and CD4 cell count at ATT initiation. The chi-square p-value for model calibration assessed using the Hosmer-Lemeshow test was 0.15. The model discrimination, measured as the area under the receiver operator characteristic (ROC) curve, was 0.78.

**Conclusion:**

The model had good internal validity, but should be validated with an independent cohort of TB-HIV co-infected patients to assess its performance before clinical or programmatic use.

## Introduction

India has the highest tuberculosis (TB) burden in the world with an estimated 2.8 million new cases in 2016 [1]. Of these cases, 87,000 (3%) were estimated to also have human immunodeficiency virus (HIV) co-infection, which is the second highest TB-HIV burden in the world after South Africa [1]. HIV co-infection in persons with TB disease increases the risk of morbidity and mortality and is one of the strongest independent predictors of unfavourable treatment outcomes (death, lost to follow-up, treatment failure) [2].

Since 2004, the World Health Organization (WHO) has recommended testing all TB patients for HIV to allow for early initiation of anti-retroviral therapy (ART) and co-trimoxazole preventive therapy (CPT), thereby reducing mortality [3]. Although, India was an early adopter of these guidelines, treatment success rates for TB patients with HIV remain lower than treatment success rates for TB patients without HIV [3]. In 2014, the treatment success rate for TB patients with HIV was 76% compared to 87% for TB patients without HIV [4]. This gap indicates opportunities to improve treatment outcomes for TB patients with HIV.

Previous studies in India have shown that the risk of unfavourable outcomes is not uniform and certain patient sub-groups experience higher unfavourable outcomes [5–9]. Age, extra-pulmonary TB, low CD4 counts (<200 cells per cubic millimeter) at the time of initiating anti-TB treatment (ATT), and history of previous TB treatment have been independently associated with unfavourable treatment outcomes [5,8]. However, quantifying the patient-specific probability of unfavourable treatment outcomes at the time of diagnosis would be helpful for both clinical and programmatic purposes. Statistical models have been used to predict the probability of future patient outcomes based on certain known characteristics and are often used in medicine and public health to guide decision-making [10]. However, such models have not been developed in India to guide clinicians and national TB programme staff managing TB-HIV co-infection.

Our study objective was to use routine surveillance data from 2015 to develop a statistical model to predict the probability of unfavourable treatment outcomes among TB patients with HIV who were registered for first-line TB treatment at RNTCP clinics in Delhi. Surveillance data from the state of Delhi were selected for analyses because Delhi has the highest TB case notification rate among all states in India (314 cases per 100,000) [11]. In addition, in 2014, patients co-infected with drug-sensitive TB and HIV in Delhi had an 11% lower treatment success rate compared to patients with drug-sensitive TB alone, suggesting a need to improve TB treatment outcomes among TB-HIV co-infected patients in Delhi [12].

## Methods

### Study setting

In Delhi, TB diagnostic and treatment services are coordinated by 25 District Tuberculosis Centres, which oversee an estimated 400 sub-district level facilities and 12 large tertiary care hospitals. HIV diagnostic and treatment services are delivered through a network of 93 Integrated Counselling and Testing Centres (ICTC) and 11 ART centres, which provide a comprehensive package of treatment and support services to people living with HIV (PLHIV). All TB patients with unknown HIV status are recommended to be tested for HIV [13]. If a TB patient tests positive for HIV, they are referred to ART centres for further evaluation and ART initiation. If TB patients are known to be HIV-positive and are already on ART, their ART is continued and managed at ART centres. HIV positive TB patients are also tested for drug resistant TB by Xpert-MTB/Rif tests and those found to be having resistance to rifampicin are treated with multidrug resistant TB treatment regimens.

Standard WHO definitions are used by RNTCP to classify TB patients and TB treatment outcomes [13,14]. The case definitions and treatment outcomes used by RNTCP are given in Table 1.

**Table 1:** Definitions and treatment regimens used under the Revised National TB Control Programme in 2015–2016.

**Disease classification** • **Pulmonary TB:** Any microbiologically confirmed or clinically diagnosed case of TB involving the lung parenchyma or the trachea-bronchial tree. ○ **Smear positive:** A new case of pulmonary TB is considered to be smear-positive if one or more sputum smear specimens at the start of treatment are positive for acid fast bacilli (AFB). ○ **Smear Negative:** A patient with symptoms suggestive of TB with at least 2 sputum smears negative for acid fast bacillus and either: a) radiographic abnormalities consistent with active pulmonary TB, as determined by the treating medical officer or b) positive culture for *Mycobacterium tuberculosis*, followed by a decision to treat the patient with a full course of anti-TB therapy. • Extra-pulmonary TB: Any microbiologically confirmed or clinically diagnosed case of TB involving organs other than the lungs such as pleura, lymph nodes, intestines, genitourinary tract, joint and bones, or meninges of the brain. **Types of TB cases** • **New**: A patient who has never had treatment for tuberculosis or has taken anti-tuberculosis drugs for less than one month. The patient can be either new smear positive, new smear negative or new extra-pulmonary TB. • **Previously treated**: A patient who has taken anti-TB treatment for more than a month from any source in the past. There are four types of previously treated cases: ○ **Relapse**: A patient declared cured of TB by a physician, but who reports back to the health service and is found to be bacteriologically positive. ○ **Treatment after loss to follow-up**: A patient who received anti-tuberculosis treatment for one month or more from any source and who returns to treatment after having defaulted, i.e. not taken anti-TB drugs consecutively for two months or more. ○ **Treatment after failure**: A smear-positive patient who is smear positive at 5 months or more after starting treatment. Failure also includes a patient who was initially smear-negative but who becomes smear-positive during treatment. ○ **Retreatment-Other**- Patients who do not fit into the above-mentioned previously treated categories. **Sputum smear status and grade (Ziehl—Neelsen Staining Method)** • **Negative**: No AFB in 100 oil immersion fields • Scanty- 1–9 AFB per 100 oil immersion fields • 1+ 10–99 AFB per 100 oil immersion fields • 2+ 1–10 AFB per oil immersion fields (in at least 50 fields) • 3+: More than 10 AFB per oil immersion field (in at least 20 fields) **TB Treatment regimen** • New TB Cases (6-month regimen): 2 months Isoniazid (H), Rifampicin (R), Pyrazinamide (Z), Ethambutol (E) + 4 months HR(E) (all drugs given thrice weekly) • Previously treated cases (8 month regimen): 2 months of HRZES (S = Streptomycin) + 1 month HRZE+ 5 months of HRE (all drugs given thrice weeks) **Anti-retroviral therapy regimens** • Patients with HIV and TB co-infection- Start ART irrespective of CD4 count and type of tuberculosis (Start ATT first, initiate ART as early as possible between 2 weeks to 2 months when TB treatment is tolerated) • Under the National Programme, co-trimoxazole preventive therapy (CPT) may be initiated in the following scenarios: ○ HIV infected adults with CD4 <250 cells/mm3 or ○ WHO clinical stage 3 or 4 irrespective of CD4 count • ART recommendations for patients who develop TB within six months of starting a first-line or second-line ART regimen- ○ First-line ART- (Zidovudine (AZT) or Tenofavir (TDF)) + Lamivudine (3TC) + Efavirenz (EFV)/ Nevirapine (NVP) ○ Second-line ART- Two Nucleoside Analogue Reverse Transcriptase Inhibitor (NRTIs) + Protease Inhibitor (PI) **Tuberculosis Treatment outcomes** • Cured- A patient who is initially smear-positive who has completed treatment and had negative sputum smears, on at least two occasions, one of which was at completion of treatment. • **Treatment completed**- Any of the following: a) a patient who was initially sputum smear-positive who has completed treatment, with negative smears at the end of the initial phase but none at the end of treatment; or b)a patient who was initially sputum smear-negative who received a full course of treatment and has not become smear-positive during or at the end of treatment; or c)a patient with extra-pulmonary TB who has received a full course of treatment and has not become smear-positive during or at the end of treatment. • **Lost to follow-up**: A patient who, at any time after registration, interrupted anti-TB drugs treatment for 2 months or more consecutively any time after starting treatment. • **Treatment failure: Either**: a) a patient who is initially smear-positive who remains smear-positive at 5 months or more after starting treatment; or b) a patient who was initially smear-negative but who became smear-positive during treatment. • **Death**: A patient who died during TB treatment, regardless of cause. • **Transferred out**: A patient who has been transferred to another Tuberculosis Unit/District and his/her treatment results are not known. • **Switched to a MDR-TB treatment regimen:** A TB patient who is on first line regimen and has been diagnosed as having DR-TB and switched to a drug resistant TB regimen prior to being declared as a treatment failure from first-line treatment.

### Study design and study population

This was a retrospective cohort study of all TB patients with known HIV-positive test results registered for first-line TB treatment in Delhi in 2015. We included both new and previously treated patients who received first-line, directly observed treatment for TB. We excluded patients with known multidrug-resistant TB, rifampicin-resistant TB, and children aged less than 15 years, in whom treatment outcomes can be impacted by factors other than HIV coinfection.

### Data collection

We abstracted data from three sources: TB registers at District TB Centres; ART registers maintained at ART centres; and individual TB treatment cards for the cohort of TB patients with HIV registered under RNTCP during 2015. We estimated the sample size for our study using the general principle of having >10 patients with unfavourable events per variable for predictive modelling to prevent the problem of overfitting [15]. The expected unfavourable event proportion was ~20% and we planned to include ten variables in our predictive model. This resulted in a sample size in excess of 450 patients was necessary in order to develop a predictive model.

### Data variables

The dependent or outcome variable for our study was dichotomised into favourable or unfavourable outcomes among patients. Favourable treatment outcomes included cure and treatment completed. Unfavourable treatment outcomes included death, loss to follow-up, treatment failure, transfer out, or a switch to MDR TB treatment.

The independent (or predictor) variables for our study included age, sex, disease classification (pulmonary versus extra-pulmonary), TB treatment category (new or previous history of ATT), sputum smear status (positive or negative), sputum smear grade, patient’s pre-treatment weight, HIV and ART status at the time of TB diagnosis, and CD4 cell count at the time of initiating the patient on ATT.

### Data validation

We cross-verified data from three sources (TB registers, ART registers, and TB treatment cards) for consistency. If data were inconsistent then data related to TB diagnosis and treatment were taken from TB registers and data related HIV were taken from ART registers.

### Data analyses

We analysed data using Stata version 12.1 (College Station, TX: StataCorp LP), and summarized categorical variables using proportions.

We used a log binomial model to estimate the crude relative risk and 95% confidence interval in order to describe the association between predictor variables and TB treatment outcomes.

To develop the predictive model we used all variables in our dataset. Only patients who had complete data on all the predictor and outcome variables were retained for model building. We initially assessed for multicollinearity between variables using Pearson’s correlation co-efficient and one of two variables that were found to be collinear (correlation co-efficient >0.7) were included. We then conducted a step-wise backward selection process to identify predictors of outcome, retaining variables with a p-value of ≤0.15. We also modelled age in years as a continuous variable or as ordinal categories (age groups). We categorised the variable sputum smear grade (which had five categories) into two categories: smear positive and smear negative/unknown and assessed for its inclusion in the model.

We used the binomial logit model to obtain the coefficients for the prediction model. We used the link test in Stata to assess specification errors in the logit link function. We also added interaction terms between various clinical and demographic variables while identifying the most suitable model. Models that did not identify specification errors were assessed for calibration using Hosmer and Lemeshow’s goodness-of-fit test and models with a chi-square p-value more than 0.05 were considered. Models were also assessed for discrimination using area under the ROC curve; models with area under the curve greater than 0.75 were considered. Akaike Information Criteria (AIC) and Bayesian Information Criterion (BIC) values were calculated for all the models that fulfilled the above criteria, and the model with the lowest AIC and BIC values was chosen as the final predictor model. The probability of an unfavourable outcome for each patient was estimated using the following binary logistic regression equation:

P(Y)=1e−(β0+β1X1+β2X2+β3X3*X4……βnXn)

where P(Y) is the probability of given outcome to be predicted, βn indicates the coefficients of the model for the X1, X2….Xn are independent variables and ‘*’ in the model indicates interaction terms between two variables.

### Ethics

We obtained approval for the study protocol from the local ethics committee of the New Delhi TB Centre (New Delhi, India); the Ethics Advisory Group of the International Union Against Tuberculosis and Lung Disease (Paris, France); and the US Centers for Disease Control and Prevention (Atlanta, GA, USA).

## Results

**Fig 1:**
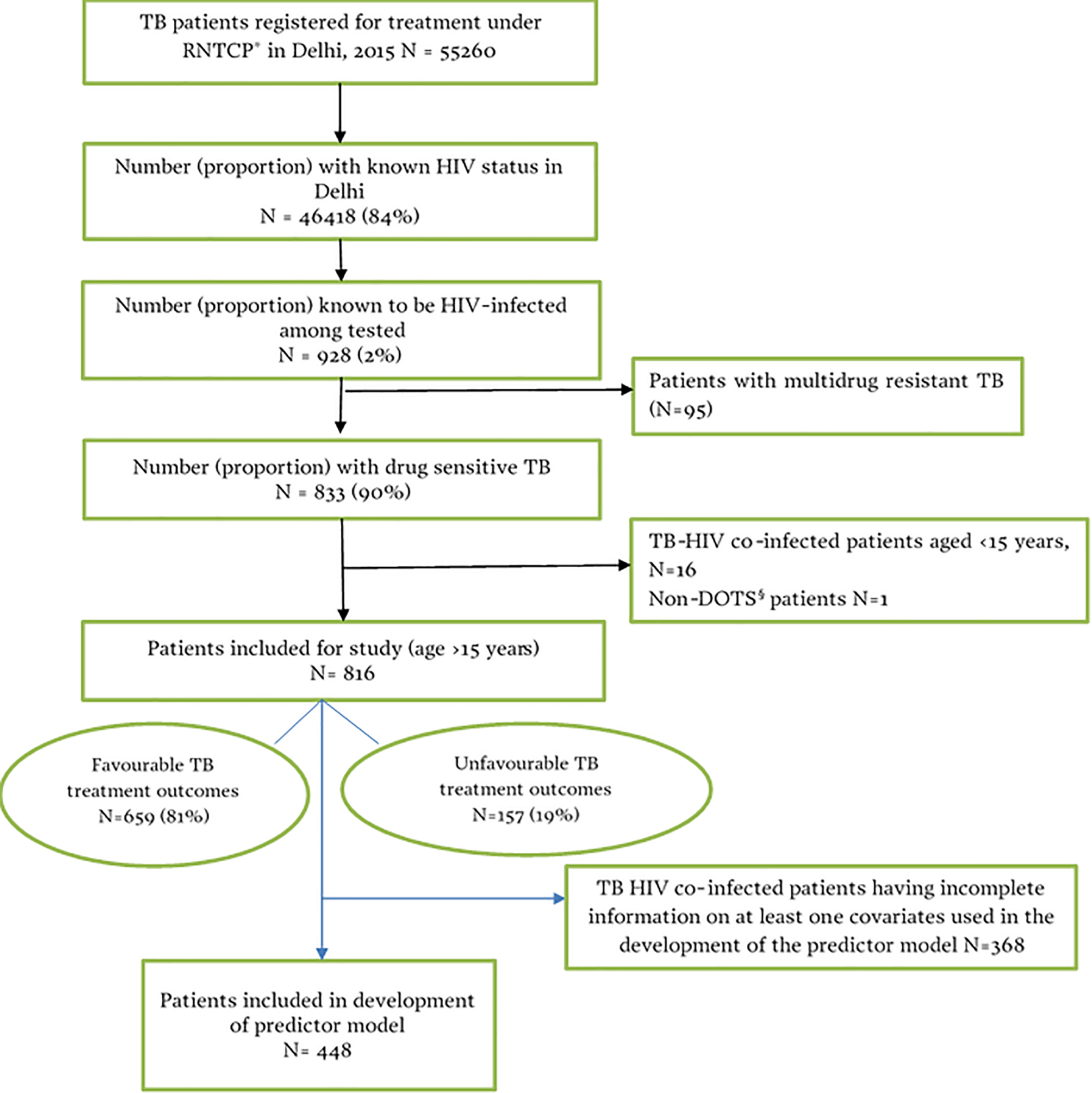
Flow diagram indicating the selection of study population. *RNTCP = Revised National Tuberculosis Programme of India; ^§^DOT = Directly Observed Therapy.

There were 55,260 TB patients registered for first-line ATT in Delhi from January through December 2015 and 928 (2%) had known HIV-positive test results (**Fig 1**)

Among these TB-HIV co-infected patients, 816 (88%) were included in our study as they had drug-sensitive TB and were ≥15 years of age. Of these, 157 (19%) had unfavourable TB treatment outcomes.

The demographic and clinical characteristics of eligible TB-HIV patients enrolled in Delhi, along with proportions of unfavourable outcomes, are presented in **Table 2**. Over two-thirds (n = 553, 67%) were aged 25–44 years of age, and male (n = 637, 78%). Almost half of these cases (n = 392, 48%) had extra-pulmonary TB, and 349 (43%) were diagnosed with HIV after presenting for TB diagnosis. Only 450 (55%) cases had a CD4 cell count recorded at TB diagnosis; of those, 342 (76%) had a CD4 cell count <350 cells per cubic millimetre. Patients who were not on ART were initiated on ART within 2–3 weeks of TB treatment. Of the 816 patients included in our study, 659 (81%) had favourable TB treatment outcomes (i.e., cured and treatment completed, **Table 2**). In bivariate analyses, age group, and sex were not associated with unfavourable TB treatment outcomes, while disease classification, history of previous ATT, smear status HIV and ART status at the time of diagnosis, and CD4 count at the time of initiating ATT were associated with unfavourable treatment outcome at p<0.15 (**Table 2**).

**Table 2:** Demographic and clinical characteristics of TB-HIV co-infected patients and bivariable analysis showing association between these characteristics with unfavourable TB treatment outcomes in Delhi, 2015 (N = 816).

Factors	n (%)	Favourable outcome (n = 659)	Unfavourable outcome (n = 157)	RR (95% Confidence interval)	p-value
	n (column %)	n (row %)	n (row %)		
**Age-group (years)**					
≥55	44 (5)	35 (80)	9 (20)	Ref	
45–54	89 (11)	73 (82)	16 (18)	0.88 (0.4–1.8)	0.730
35–44	243 (30)	207 (85)	36 (15)	0.72 (0.4–1.4)	0.335
25–34	310 (38)	255 (82)	55 (18)	0.87 (0.4–1.6)	0.658
15–24	130 (16)	89 (68)	41 (32)	1.5 (0.8–3.0)	0.182
**Sex**					
Female	175 (21)	144 (82)	31 (18)	Ref	
Male	637 (78)	512 (80)	125 (20)	1.1 (0.8–1.6)	0.573
Transgender	4 (0.5)	3 (75)	1 (25)	1.4 (0.3–8.0)	0.700
**Type of patient**					
New	543 (67)	455 (84)	88 (16)	Ref	
Previously treated	273 (33)	204 (75)	69 (25)	1.6 (1.2–2.1)	0.002
**Disease type**					
Extra-pulmonary	392 (48)	349 (89)	43 (11)	Ref	
Pulmonary	398 (49)	292 (73)	106 (27)	2.4 (1.8–3.4)	<0.001
Both	26 (3)	18 (69)	8 (31)	2.8 (1.5–5.3)	0.002
**Sputum smear grade**					
Negative	226 (28)	186 (82)	40 (18)	Ref	
1+	67 (8)	45 (67)	22 (33)	1.9 (1.2–2.9)	0.006
2+	33 (4)	23 (70)	10 (30)	1.7(0.95–3.0)	0.073
3+	63 (8)	37 (59)	26 (41)	2.3 (1.6–2.5)	<0.001
Scanty	17 (2)	14 (82)	3 (18)	0.99 (0.3–2.8)	0.996
Positive, Unknown	18 (2)	13 (72)	5 (28)	1.6 (0.5–1.1)	0.276
**CD4 cell count at ATT initiation (cells/mm**^**3**^**)**					
>500	47 (6)	45 (96)	2 (4)	Ref	
350–499	61 (7)	54 (89)	7 (11)	2.7(0.6–12)	0.232
200–349	117 (14)	96 (82)	21 (18)	4.2 (1.0–17)	0.041
51–199	178 (22)	142 (80)	36 (20)	4.8 (1.2–19)	0.022
<50	47 (6)	33 (70)	14 (30)	7.0 (1.7–29)	0.007
Not recorded	366 (45)	289 (79)	77 (21)	5.0 (1.3–19)	0.026
**HIV Status at TB diagnosis**					
Known HIV, on ART	263 (32)	215 (82)	48 (18)	Ref	
Known HIV, not on ART	37 (5)	23 (62)	14 (38)	2.07 (1.3–3.0)	0.003
HIV diagnosis after TB diagnosis	349 (43)	299 (86)	50 (14)	0.78 (0.5–1.1)	0.195
Known HIV, ART status not recorded at diagnosis	167 (20)	122 (73)	45 (27)	1.48 (1.03–2.1)	0.032

### Predictor model

Of 816 patients included in our study, 448 (55%) had complete information on age, sex, disease classification, history of previous ATT, HIV status, ART status, and CD4 cell count at ATT initiation. These patients were included in our model. There were no statistical differences in clinical characteristics (disease classification, type of TB, sputum smear status and the outcome) among patients included and excluded in the model **(Table 3)**.

**Table 3:** Comparison of clinical characteristics and treatment outcomes of TB-HIV patients with complete data vs incomplete data on co-variates* included for building a predictive model for unfavourable model in Delhi.

Clinical characteristics	Total N = 816	Patients with complete data* N = 448		Patients with incomplete data** N = 368		Chi^2^ p-value
**Sputum Smear**	** **	**n**	**%**	**n**	**%**	** **
Positive	198	108	24	90	24	0.908
Negative	618	340	76	278	76	
**Disease classification**						
Pulmonary	392	219	49	173	47	0.614
Extra-Pulmonary	398	217	48	181	49	
Both	26	12	3	14	4	
**TB treatment category**						
New	543	311	69	232	63	0.055
Previously treated	273	137	31	136	37	
**Outcome**						
Favourable	659	370	83	289	79	0.144
Unfavourable	157	78	17	79	21	

* Age and sex were not independently associated with the outcome (as shown in Table 2) and therefore they have not been included in this table here; patients with complete data were included for developing a prediction model.

** missing/incomplete data were predominantly due to CD4 cell counts and ART status of these patients.

The step-wise backward selection process to identify the most relevant predictors of outcome (retaining the variables with a p-value of ≤0.15) identified sputum smear grade [or sputum smear status (positive or negative)], previous history of ATT, disease classification (pulmonary versus extra-pulmonary), HIV and ART status at the time of TB diagnosis, and CD4 cell count at the time of initiating the patient on ATT as the most important predictors for unfavourable outcome. We used multiple combination/iterations of these variables to identify the most appropriate model. While assessing the models, we added age and sex into the model as these are programmatically relevant and this improved the model performance and therefore these two variables were retained. We also added interaction terms between demographic and clinical characteristics (age and sex; sputum smear status and type of TB; HIV status at TB diagnosis and CD4 cell category) alone and in combination. Models that had p-values >0.05 for model calibration (Hosmer-Lemeshow test with 10 groups), relatively lower levels of AIC, BIC values and relatively higher value for model discrimination (areas under the ROC curve for sensitivity and 1 minus specificity) were considered to be relatively better performing models. The various combinations/iterations of the variables that we used are shown in **Table 4** and the footnote of the table provides the variable properties. The model that performed best in our iterations contained the following variables: sputum smear grade; new/previous history of ATT; disease classification (pulmonary versus extra-pulmonary); HIV status, ART status, and CD4 cell count at the time of TB diagnosis; sex and age (with interaction terms between age and sex; sputum smear status and type of TB; HIV status at TB diagnosis and CD4 cell category). The coefficients of the prediction model that we selected are shown in **Table 5**.

**Table 4:** Variable combinations/iterations used for selecting the prediction model and its performance values for selecting the most appropriate model for predicting unfavourable outcomes in TB-HIV patients enrolled for TB treatment in 2015 in Delhi.

Variables included in the model *(Please see footnote to this table to know the variables)*	p-value (model calibration)	Akaike Information Criteria (AIC)	Bayesian Information Criterion (BIC)	Area under ROC (model discrimination)
PTS, CAT, DISCLAS, HIVSTAT, CD4 (no interaction)	0.716	387.070	407.594	0.702
PTS, CAT, DISCLAS, HIVSTAT, CD4 (interaction between PTS&CAT)	0.691	386.529	407.053	0.708
PTS, CAT, DISCLAS, HIVSTAT, CD4, Sex, Age (no interaction)	0.644	378.468	398.959	0.711
PTS, CAT, DISCLAS, HIVSTAT, CD4cat3, Sex, Age (no interaction)	0.432	379.485	399.976	0.704
PTS, CAT, DISCLAS, HIVSTAT, CD4cat3, (interaction between PTS & CAT)	0.458	387.037	407.561	0.699
PTS, CAT, DISCLAS, HIVSTAT, CD4cat3, Sex, Age (with interaction between PTS &CAT)	0.408	379.051	399.541	0.706
PTS, CAT, DISCLAS, HIVSTAT, CD4Cat3, Sex, Agegrp (interaction between PTS&CAT; Sex & Agegrp)	0.273	366.483	386.951	0.745
PTS, CAT, DISCLAS, HIVSTAT, CD4cat3, Sex, Agegrp (interaction between PTS&CAT)	0.574	373.084	393.574	0.726
PTS, CAT, DISCLAS, HIVSTAT, CD4cat3 Sex Agegrp (interaction between PTS&CAT; HIVSTAT & CD4cat, Sex & Agegrp)	0.252	361.194	381.662	0.747
Grade, CAT, DISCLAS, HIVSTAT, CD4cat3, Sex, Age (no interaction)	0.342	374.241	394.731	0.726
Grade, CAT, DISCLAS, HIVSTAT, CD4cat, Sex, Agegrp (no interaction)	0.789	364.750	385.241	0.749
Grade, CAT, DISCLAS, HIVSTAT, CD4cat, Sex, Agegrp (interaction between Sex & Agegrp)	0.495	351.263	371.731	0.775
Grade, CAT, DISCLAS, HIVSTAT, CD4cat, Sex, Agegrp (interaction between HIVSTAT & CD4cat)	0.667	359.594	380.084	0.758
Grade, CAT, DISCLAS, HIVSTAT, CD4cat, Sex, Agegrp (interaction between Grade & CAT)	0.302	356.281	376.703	0.756
Grade, CAT, DISCLAS, HIVSTAT, CD4cat, Sex, Agegrp (interaction between Grade & CAT, Sex & Agegrp)	0.547	347.719	368.119	0.781
Grade, CAT, DISCLAS, HIVSTAT, CD4cat, Sex, Agegrp (nteraction between Grade& CAT, HIVSTAT & CD4cat)	0.442	350.893	371.316	0.765
Grade, CAT, DISCLAS, HIVSTAT, CD4cat, Sex, Agegrp (interaction between HIVSTAT & CD4cat, Sex & Agegrp)	0.495	351.263	371.731	0.775
Grade, CAT, DISCLAS, HIVSTAT, CD4cat, Sex, Agegrp (interaction between Grade &CAT, HIVSTAT& CD4cat, Sex & Agegrp)	0.149	341.794	362.194	0.783

Footnote providing variables and its definitions

PTS = Pre-treatment sputum smear [Categorical variable: 2 categories (Positive, Negative/Unknown)]; Grade = Sputum smear grade {Categorical variable: 7 categories [(smear negative; smear positive (grade scanty); smear positive (grade 1+); smear positive (grade 2+); smear positive (grade 3+); No sputum smear; grade unknown)]}; CAT = TB treatment category [Categorical variable: 2 categories (New, Previously treated)]; DISCLAS = Disease Classification [Categorical variable: 3 Categories (Pulmonary, Extra-pulmonary, Both)]; HIVSTAT = HIV diagnosis at the time of TB diagnosis [Categorical variable: 3 Categories (Known HIV seropositive on ART; Known HIV Seropositive not on ART; HIV diagnosed after TB diagnosis); CD4 = CD4 cell count in mm3 [Integer variable: Values from 0–2000); CD4cat = CD4 cell categories cells/mm3 [Categorical variable: 5 categories (> = 500, > = 350-<500; > = 200-<350; > = 50 to <200; <50)]; CD4cat3 = CD4 cell categories cells/mm3 [Categorical variable: 3 categories (> = 350, > = 200-<350; <200)]; Sex = Sex of the patient [Categorical variable: 2 Categories (Male, Female)]; Age = Age of the patient (Integer variable: Values from 15–99]; Agegrp = Age categories [Categorical variable: 4 categories (15–24; 25–35; 35–44; > = 45)]// based on WHO TB reporting weight bands

**Table 5:** Coefficients (log of odds ratios), its standard errors, p-value and odds ratios (95% CI) of the prediction model for unfavourable TB treatment outcomes among TB-HIV co-infected patients enrolled for TB treatment in Delhi, 2015 (n = 448).

	N	Co-efficient	Robust Standard errors *	p-value	Odds ratio (95% CI)
**TB treatment category and smear grade**					
**New**					
Smear Negative	87	Reference			
Smear Positive (grade Scanty)	6	0**	-	-	-
Smear positive (grade 1+)	22	1.754	0.584	0.003	5.78 (1.84–18.16)
Smear Positive (grade 2+)	15	0.990	1.088	0.362	2.69 (0.32–22.71)
Smear Positive (grade 3+)	14	0.524	0.720	0.466	1.69 (0.41–6.93)
New TB (no sputum smear)	154	0.515	0.697	0.459	1.67 (0.43–6.56)
Smear positive (grade unknown)	13	0.002	0.490	0.996	1.00 (0.38–2.61)
**Previously treated**					
Smear Negative	46	0.303	0.436	0.487	1.35 (0.57–3.18)
Smear Positive (grade Scanty)	3	1.557	1.596	0.329	4.74 (0.20–108.42)
Smear positive (grade 1+)	10	0.480	0.967	0.619	1.61 (0.24–10.76)
Smear Positive (grade 2+)	6	0.052	1.697	0.975	1.05 (0.04–29.31)
Smear Positive (grade 3+)	17	2.021	0.896	0.024	7.54 (1.30–43.73)
Retreatment TB (no sputum smear)	53	0.902	0.545	0.098	2.46 (0.84–7.17)
Smear positive (grade unknown)	2	3.413	1.280	0.008	30.37 (2.47–373.47)
**Disease classification**					
Extra-pulmonary	219	Reference			
Pulmonary	217	0.607	0.218	0.005	1.83 (1.19–2.81)
Both	12	1.769	0.518	0.001	5.86 (2.12–16.19)
**HIV diagnosis status at TB diagnosis and CD4 Cell count (cells/mm**^**3**^**)**					
Known HIV seropositive on ART with CD4 cell count					
> = 500	22	reference			
> = 350-<500	25	2.091	1.439	0.146	8.09 (0.48–135.90)
> = 200-<350	37	1.816	1.117	0.104	6.14 (0.68–54.93)
>50-<200	70	2.794	1.358	0.04	16.35 (1.14–234.1711)
<50	10	3.657	1.788	0.041	38.76 (1.16–1289.61)
Known HIV seropositive not on ART with CD4 cell count					
> = 500	1	0**	-	-	
> = 350-<500	2	3.978	1.993	0.046	53.44 (1.07–2656.37)
> = 200-<350	3	4.189	1.059	0	65.98 (828–525.49)
>50-<200	5	1.938	1.630	0.234	6.94 (0.28–169.41)
<50	3	3.895	1.711	0.023	49.20 (1.72–1406.47)
HIV diagnosed after TB diagnosis with CD4 cell count					
> = 500	25	0.053	1.619	0.974	1.05 (0.044–25.20)
> = 350-<500	34	0.916	1.036	0.376	2.49.(0.32–19.02)
> = 200-<350	75	2.183	1.213	0.072	8.88 (0.82–95.72)
> = 50-<200	103	1.916	0.750	0.011	6.79–1.56–29.51)
<50	33	2.638	1.125	0.019	13.99 (1.54–126.84)
**Sex & Age**					
Female (age in years)					
15–24	8	Reference			
25–34	37	2.242	0.877	0.011	9.41 (1.68–52.55)
35–44	28	0.56	0.822	0.495	1.75 (0.34–8.77)
= >45	11	1.502	0.782	0.055	4.49 (0.97–20.78)
Male (age in years)					
15–24	44	2.364	0.779	0.002	10.63 (2.30–48.99)
25–34	134	1.009	0.661	0.127	2.74 (0.75–10.02)
35–44	118	1.065	0.629	0.091	2.90 (0.84–9.96)
= >45	65	1.378	0.880	0.117	3.96 (0.70–22.25)
**Constant/intercept value**		-6.055	1.646	0	0.002 (0.00–0.06)

* adjusted for 6 zones of Delhi

** coefficient same as the reference category

**Fig 2:**
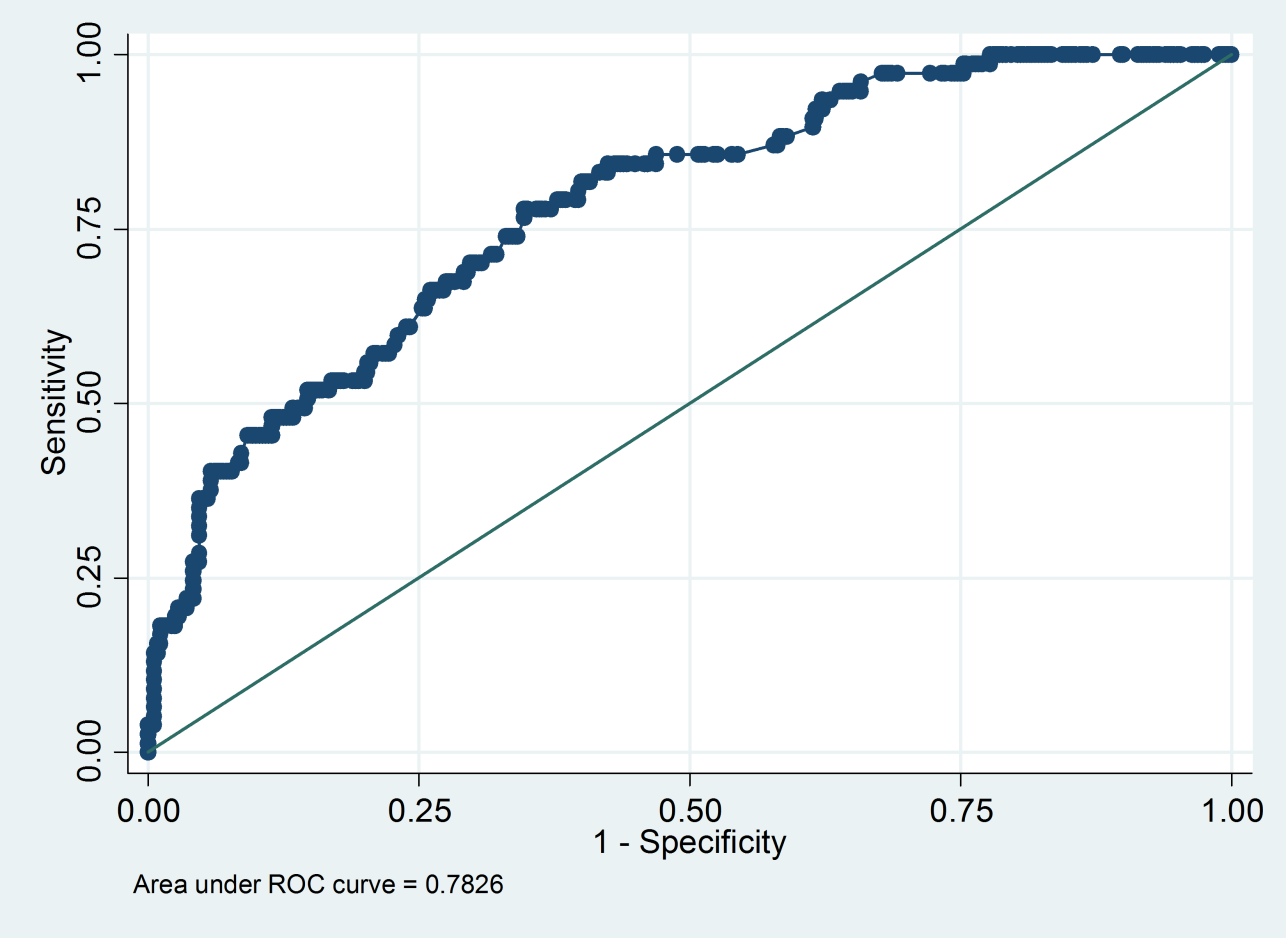
Receiver operating characteristics curve and the area under the curve for various sensitivity and specificity values of the prediction model developed for identifying TB HIV patients with unfavourable TB treatment outcomes under RNTCP in Delhi, India, 2015.

**Fig 3:**
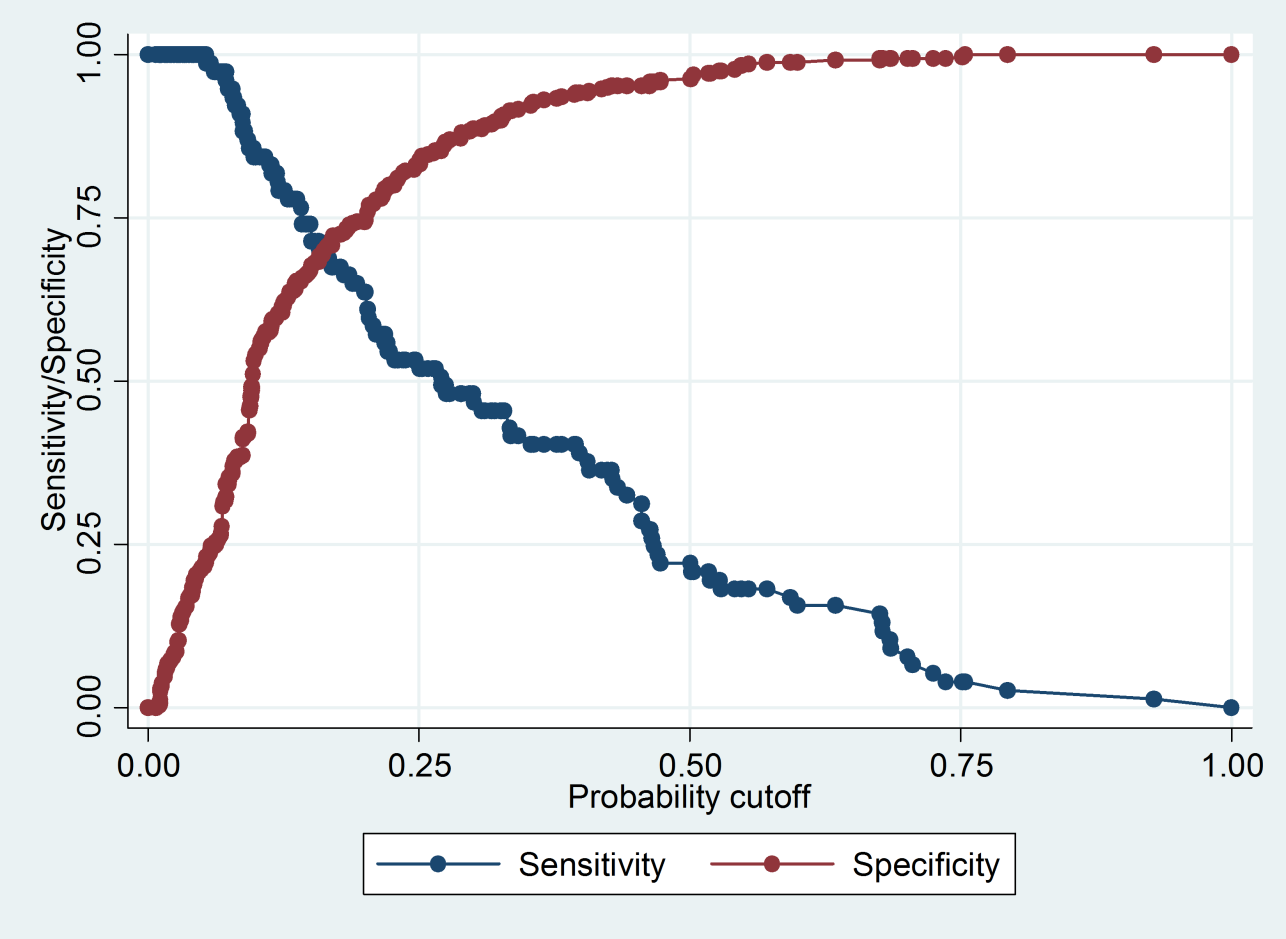
Sensitivity and specificity of various cut-off values of predicted probabilities derived from a prediction model for identifying unfavourable TB treatment outcomes in TB HIV patients under RNTCP in Delhi, 2015.

The chi-square p-value for the model calibration using the Hosmer-Lemeshow test with 10 groups for this model was 0.14, and the discrimination of the prediction model, measured as the area under the receiver operator characteristic (ROC) curve, was 0.78 (**Fig 2**), an indication that the model had good discriminatory and identifying ability to identify with unfavourable outcomes. The sensitivity and specificity for identifying patients with adverse outcomes at various cut-off values of predicted probabilities derived from the prediction model are shown in **Fig 3**.

## Discussion

The model developed in this study was used to predict the probability of unfavourable outcomes in TB patients with HIV at the time of initiating ATT in Delhi and had good internal validity.

The results of this study should be interpreted in the context of its limitations. First, this model was developed using secondary data collected under routine programmatic conditions; therefore, the validity of this model was dependent on the accuracy and completeness of the data recorded. Although our study had substantial missing data, there are data monitoring systems in place [16], and therefore errors in recording are likely to be random and would not substantially influence model parameters. Additionally, comparison of data for patients included and excluded from the prediction model appear similar (Table 3), further suggesting that data were missing at random and therefore should not substantially influence the model. Second, we only used variables that are routinely collected by TB and HIV surveillance systems at the time of diagnosis. As such, we were unable to include variables that are known to be associated with unfavourable outcomes, such as HIV viral load, TB drug resistance status, other co-morbidities, homelessness, and socio-economic status [17] because these variables were not recorded by the surveillance systems. The extent to which model fit could have been improved by inclusion of such unmeasured variables is an area for future research. Third, we used data from patients who were registered for TB treatment under the national TB programme. Approximately 10% of patients diagnosed with TB in India do not receive treatment due to pre-treatment loss to follow-up, while a large number of TB patients receive treatment from health care providers in the private sector who are outside the national TB programme [18,19]. In addition, our model may not reflect the probabilities of unfavourable outcomes for TB-HIV patients outside Delhi or in other states of India or in other calendar years. Fourth, we have used various iterations of multivariable logistic regression for developing the prediction model manually (**Table 4**). There are several other emerging newer ways of model building using sophisticated machine learning techniques which automates the process [20–22]. These newer techniques may have resulted in identifying models that have a relatively better fit. This is an area for future research. Finally, the model’s applicability may change in the future when case definitions and management protocols may change.

Despite these limitations, developing prediction models from data that are routinely collected is a first step towards informing clinicians and programme managers about patients at risk of unfavourable outcomes. Previous studies in India have looked at patients’ demographic and clinical characteristics in isolation and have described whether these variables are independently associated with unfavourable outcomes. By contrast, our study derived a mathematical equation, which can be used to estimate the probabilities of unfavourable outcomes for patients at the time of ATT initiation. This is an improvement over previous studies as it allows for a more tailored approach to care, such as more frequent monitoring of patients or more focused nutrition supplementation. That said, the model developed is not yet ready to be used in clinical or public health practice. As a next step, the model needs to be validated on an independent sample of patients to assess the model performance. This process is also known as “validation” of model using an external sample [23] and could be done using a cohort of TB-HIV patients enrolled in the year 2016. Depending on the performance of the model in this external cohort of patients, the model may be considered for clinical or programmatic use.

In addition to the model, there were notable findings from this study that have important public health implications. First, this study shows that in 43% of the cases, HIV was detected subsequent to TB diagnosis, suggesting that routine HIV testing among TB patients remains important for identification of new HIV cases. However, 16% of TB patients registered for treatment under RNTCP in Delhi during 2015 did not have a documented HIV test result, indicating missed opportunities to detect new HIV cases. These findings call for re-examining strategies for early detection of HIV in key populations to allow for early initiation of ART and early initiation of either ATT or isoniazid preventive therapy to prevent TB altogether.

Second, the overall proportion of patients with favourable TB treatment outcomes in this cohort was 81%, higher than in previous studies conducted in southern India and in tertiary care centers in northern India, where treatment success ranged between 66% and 77% [8,9]. This could be because our study occurred in an era in which ART is widely available compared to earlier studies conducted when ART was not as readily available to patients.

**In conclusion**, approximately, one in five TB-HIV co-infected patients in Delhi enrolled for treatment under the RNTCP in 2015 had an unfavourable TB treatment outcome. A statistical model with good calibration and discrimination was developed using demographic and clinical data routinely collected by the RNTCP at the time of enrolling patients for TB treatment. This model should be externally validated across multiple years of RNTCP data, then with an independent cohort of TB-HIV patients to assess its performance in Delhi and other areas of India before it is used in clinical and/or programmatic practice to predict unfavourable outcomes among patients co-infected with TB and HIV.
